# Gabapentin Determination in Human Plasma and Capsule by Coupling of Solid Phase Extraction, Derivatization Reaction, and UV-Vis Spectrophotometry 

**Published:** 2013

**Authors:** Maryam Kazemipour, Iman Fakhari, Mehdi Ansari

**Affiliations:** a *Department of Chemistry, Faculty of Sciences, Kerman Branch, Islamic Azad University, Iran.*; b *Herbal and Traditional Medicines Research Center, Kerman University of Medical Sciences, Kerman, Iran. *

**Keywords:** Gabapentin, Spectrophotometry, Derivatization, SPE, Plasma, Vanillin

## Abstract

Gabapentin is an anticonvulsant widely used in the treatment of epilepsy. No peculiar chromophore is available on the gabapentin moiety for direct analysis by absorption spectrophotometry. A sensitive spectrophotometric method for the determination of gabapentin in bulk, pharmaceutical formulations and human plasma has been developed. In this method, gabapentin directly derivatized with vanillin and analyzed without any extraction in bulk and pharmaceutical dosage form and in plasma samples, it was extracted with a reversed-phase solid-phase extraction (SPE) cartridge followed by derivatization with vanillin. Analysis was performed by a spectrophotometer system. The quantitation limit of gabapentin in human plasma was 0.8 mg/L. The method was linear over the concentration range of 10.0–90.0 mg/L and 0.8–10.0 mg/L for pharmaceutical dosage form and plasma, respectively. The method was precise (relative standard deviation, RSD <1.20%) and accurate (relative mean error <5.5%) for both pharmaceutical dosage form and plasma samples. Mean absolute recoveries were 94.5% for plasma.

## Introduction

Gabapentin (GBP), 1-(amino methyl) cyclohexane acetic acid, is a structural analogue of the inhibitory neurotransmitter *g*-amino butyric acid. In recent years, gabapentin has been used widely as an adjunct for the treatment of acute postsurgical pain. Several meta-analyses have confirmed the efficacy of gabapentin in reducing postoperative opioid use and pain associated with heroin withdrawal (1, 2). As GBP has no significant ultraviolet, visible or fluorescence absorption, analysis of the drug has been achieved following its derivatization to produce a chromophore, detectable by fluorescence or UV detectors (3). Several methods for the analysis of gabapentin in pharmaceutical formulations, including UV-Vis spectrophotometry (4-6), spectrofluorometry, colorimetry, and CE have been reported (7). Various analytical methods for therapeutic monitoring also have been reported in the literature for the quantitative determination of gabapentin in human plasma or serum using GC (8, 9), CE (10, 11), and HPLC (12-16). The GC and CE assays are likely to be limited by routine availability of these techniques. The chromatographic technique most used is HPLC analysis that needs an extraction and derivatization step before quantitative determination. Most HPLC techniques involve derivatization with 2, 4, 6-trinitrobenzene-sulphonic acid (14), phenylisothiocyanate (17) with spectrophotometric detection, while o-phthaldialdehyde (18) and dansyl chloride (19) in spectrofluorimetric detection. In CE methods, fluorescamine (20) and 6-carboxyfluorescein succinimidyl ester (21) are used to obtain fluorophore. Methods with tandem have been reported for the determination of GBP in human biological fluids that is quite limited (22).

To the best of our knowledge, there are no spectrophotometric methods in the literature for the analysis of gabapentin in human plasma. In the present study, a spectrophotometric method for the determination of GBP in pharmaceutical formulation and coupled with SPE for determination of GBP in plasma is described. The procedure is based on the off-line derivatization of the drug with vanillin. The method was used successfully to evaluate the potency of marketed GBP capsules as well as the concentration of GBP in human plasma.

## Experimental

*Reagents*

GBP and a generic capsule produced by Alhavi pharmaceutical Co. were kindly donated by Alhavi Pharmaceutical Co., Iran. Neurontin^®^ capsule produced by Pfizer were purchased from the local market. Citric acid, sodium hydroxide, sodium hydrogen phosphate, vanillin, ninhydrin, hydrochloric acid, methanol, boric acid, dichloromethane, chloroform, potassium chloride, and sodium 1,2-naphthoquinone-4- sulfonate (NQS). All chemicals were of analytical reagent grade made by Merck (Darmstadt, Germany) purchased from the local market. In all experiments deionised Milli-Q water was used. Blood plasma was obtained from healthy human volunteers and collected into tubes treated with disodium EDTA as an anticoagulant. Plasma samples were stored at approximately −20 ^o^C until they were analyzed.

*Preparation of standard solutions*

Stock standard solution of gabapentin of 1000.0 mg/L was prepared in water. Before analysis, the required concentrations of GBP (10–90 mg/L) were prepared by mixing appropriate volume of stock standard solutions, and diluting with water.

*Preparation of McIlvaine buffer*

McIlvaine buffer (23) was prepared by mixing 35.5 mL of 0.2 M disodium hydrogen phosphate with 64.5 mL of 0.1 M citric acid and pH was adjusted to 7.5 with 0.1 N sodium hydroxide. 

*Preparation of duquenois reagent*

Duquenois reagent (24) was prepared by mixing 2 g of vanillin with 0.3 mL of acetaldehyde and the volume was completed to 50 mL with ethyl alcohol. The reagent should be prepared daily and stored in a dark place.

*Derivatization procedure*

Into 10 mL measuring flasks, different aliquots of drug solution (0.2-0.9 mL) were transferred to provide final concentration range 10-90 mg/L. To each flask, 1 mL of Duquenois reagent and 1 mL of McIlvaine buffer of pH 7.5 were successively added. During this stage, flasks were kept in a dark place and shook intermittently. The volume was made up to the mark with distilled water and the absorbance was measured against a reagent blank in the range of 380 to 450 nm. The calibration graph was prepared by plotting maximum absorbance vs. concentration of GBP.

*Optimization of reaction temperature*

Standard solutions of GBP 50 mg/L in different flasks were prepared and each one was held at 30, 40, 50, 60, and 70 ^o^C. After the completion of reaction, the volume of the flask made to the mark, and their absorbances were measured against a reagent blank in the range of 380 to 450 nm.

*Time of reaction*

Effect of time on the completion of reaction based on chromophore formation was studied at different times, including 30, 60, 90, 120, and 150 min after the addition of derivation reagent. To perform this stage, at least five flasks containing standard solution of GBP 50 mg/L were used and operated. Then at each above-mentioned time, one of the flasks was made to volume, and the solution absorbance was measured against a reagent blank in the range of 380 to 450 nm. 

*Reaction pH*

In this stage, as in the previous sections, standard solutions of GBP 50 mg/L were prepared, but the buffer with pH_s_ of 5.5, 6.5, 7.5, 8.5 and 9.5 were added. After the completion of reaction, the volume of the flask made to the mark, and their absorbances were measured against a reagent blank in the range of 380 to 450 nm. 

*Validation*

*Selectivity*

*Interferences from excipients*

In order to evaluate interferences of excipients such as talc, lactose, starch, microcrystalline cellulose, gelatin, and magnesium stearate, which are usually present in the capsule or tablet formulations, a mixture of the excipients by percent around the usual amount in the formulations were prepared, then the mixture was dissolved in water to obtain a solution with around the same concentration as real samples and the derivatization procedure was applied to the solution (0.5 mL), and then its spectrum was obtained in the range of 200 to 600 nm to find any probable interferences from excipients.

*Interferences from degradation products in stress condition*

Degradation products of GBP may potentially interfere in the analysis of parent molecule. To evaluate probable interferences from them, the working standard solutions of 50 mg/L of GBP were stressed by high temperature and refluxed in HCl 0.1 N and NaOH 0.1N separately, as the chemical stress condition for four hours. GBP was assayed by previously described method in samples withdrawn from the reflux media at times 0 to 4 h. Degradation of GBP in such a condition that may produce degradation products faster than normal conditions, spectra with the same overall shape as GBP, but with lower absorbance in each wavelength may interpret as selectivity of the method.

*Linearity*

The linearity of the method was evaluated by a calibration curve in the range of 10–90 mg/L of the drug (n = 5). The samples were assayed using the method described above. Calibration graphs were prepared by plotting the absorbance of GBP reaction product versus the drug concentrations with least-squares linear regression analysis. The quality control (QC) samples were separately prepared in water at the concentrations of 10, 50 and 90 mg/L.

*Accuracy*

The mean recovery of GBP at three QC levels (10, 50 and 90.0 mg/L) (n = 3) was calculated by comparing the concentration obtained from the drug supplemented water solution to the actually added concentration. 

*Precision*

Intra-day and inter-day precision were determined in standard samples by determining QC samples at three concentration levels (10, 50 and 90.0 mg/L). For intra-day assay precision, six replicates of samples at each concentration were assayed all at once within a day. The inter-day assay precision was determined by analysing samples on six different days. Six replicates at each concentration were assayed per day.

*LOD and LOQ*

The limit of detection (LOD) is the minimum quantity or concentration that can be distinguished from zero. The limit of quantification (LOQ) is the minimum quantity or concentration that can be evaluated with a certain precision. In this study, 3 and 10 -criteria were applied for the calculation of LOD and LOQ values that were evaluated by standard procedures.

*Stability of the method*

Stability of the samples and sample preparations were tested by analysing standard solutions and sample preparations stored at the ambient temperatures and protected from light by covering the bottles with aluminum foil, at 1, 2, 4, 8, and 16 days after preparation. 

*Pharmaceutical dosage form analysis*

The contents of capsules (Alhavi and Pfizer product) labeled to contain 100 mg GBP removed, as completely as possible, and transferred an accurately weighed portion of the powder, equivalent to about 25 mg of GBP, to a 25 mL volumetric flask, and dissolved in the water and made to volume. This stock solution of GBP was used as the standard stock solution to prepare solutions acclaimed to contain 50 mg/L of GBP. Then according to the developed method, the solution was analyzed for its GBP content. 

*Plasma sample analysis*

The developed method was applied for determination of GBP in plasma. To do this, it needed to concentrate and purify the plasma samples for its GBP content. Concentration and purification were performed on a C18- SPE cartridge preconditioned by passing 1 mL methanol and 0.5 mL of 1 M monobasic sodium phosphate, consecutively. Standard spiked plasma solutions (1-5 mg/L) of GBP then were passed through the conditioned cartridge. The cartridge was washed by 0.25 mL monobasic sodium phosphate and 1 mL HCl 0.1 N, consecutively. GBP was eluted by 1 mL methanol. These solutions were used to determine their GBP contents according to the developed method. Drug-free plasma was used to evaluate the interfering effect of plasma components on the analysis. 

## Results and Discussion

*Optimisation of the derivatization reaction*

**Figure 1:**
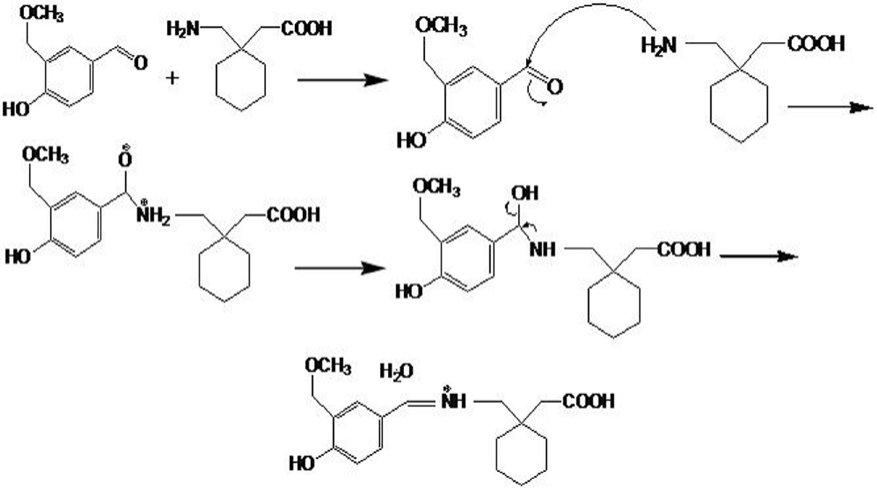
Recommended mechanism for vanillin and gabapentin reaction to produce a chromophore

Gabapentin shows weak absorption in UV range and cannot be detected by UV detectors. Derivatization by chromophoric reagents increases the sensitivity of gabapentin detection. Different derivation reagents have been reported to be used for gabapentin (14, 19-22). Gabapentin contains a primary aliphatic amino group, which is known to react with many color reagents such as vanillin, ninhydrin and p-benzoquinone (24). In this study, vanillin, which contains an aldehyde group that could react with the primary amino group of gabapentin via the condensation mechanism to give colored product, was used. The mechanism of reaction is proposed in Figure 1. 

**Figure 2:**
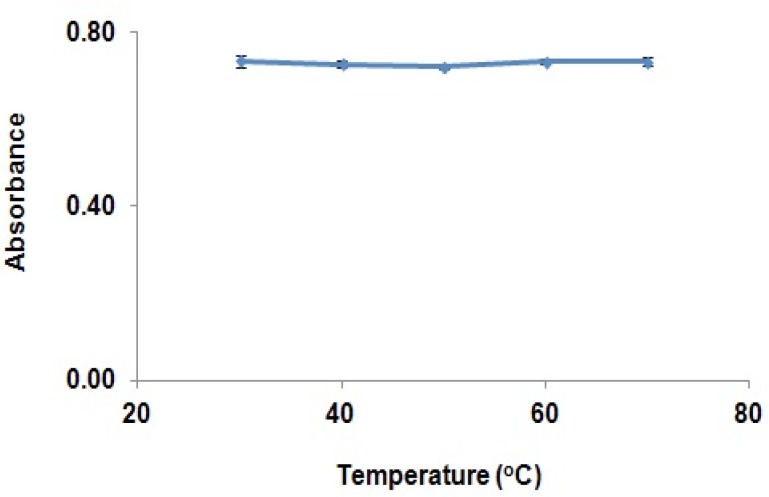
Effect of temperature on completion of GBP derivatization (absorbance of derivatised GBP in the solution at various temperatures (30 to 70^o^C)).

As it can be seen in the figure, GBP and vanillin react to produce a chromophore with acceptable absorbance at 402 nm. In order to optimise the derivatization conditions for GBP, main parameters affecting the derivatization of GBP were briefly studied, including reaction time, reaction temperature, and pH of reaction. The effects of the parameters were evaluated and optimized based on the absorbance at peak wavelengths. The effects of various reaction temperatures on derivation of GBP in Figure 2, show that equilibrium formation of the derivative is not changed significantly in the temperature range used in this study. 

**Figure 3:**
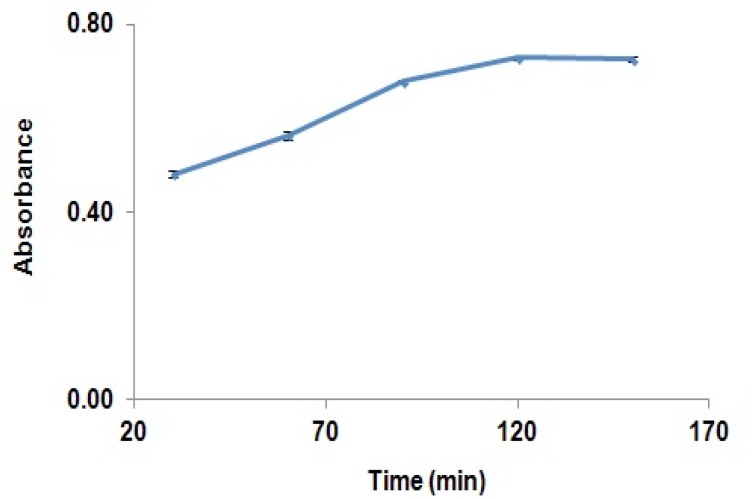
Effect of time on completion of GBP derivatization reaction, (absorbance of derivatised GBP in the solution at different times (30 to 150 min)).

Therefore, derivatization reaction can be performed at the ambient temperatures. The effects of derivatization reaction time on derivation GBP have been shown in Figure 3. 

**Figure 4:**
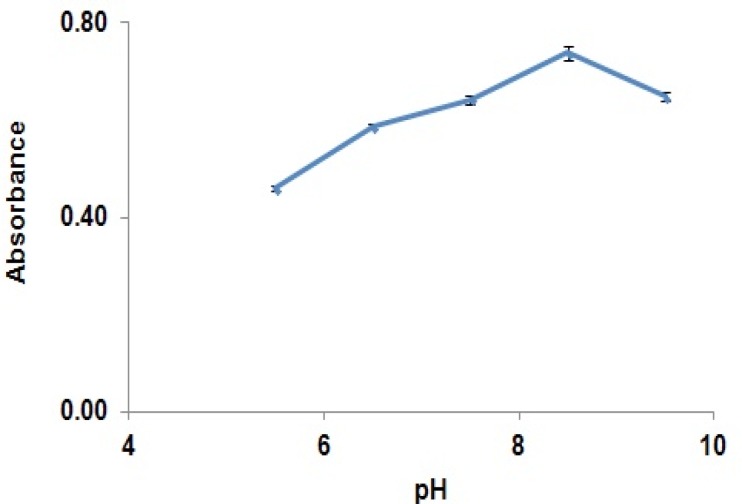
Effect of pH of the reaction medium on completion of GBP derivatization reaction (absorbance of derivatised GBP in the solution at different pHs (5.5 to 9.5)).

Absorbance of the reaction yield is significantly increased from 30 min to 120 min, while after this time, the absorbance was remained constant. Results showed that solutions were stable for at least two hours after completion of the reaction. The effect of pH on the derivatization reaction yield has been shown in Figure 4. 

It reveals that increasing pH from 5 to 8.5 would increase reaction yield and after that, reaction yield was decreased. Based on the optimized derivatization condition, pH 8.5 was selected. 

*Method validation*

The method was tested for linearity, selectivity, precision, LOD, and LOQ. By using the above spectrophotometric procedure, linear regression equations were obtained. The regression plots showed that there was a linear dependence of the absorbance on the concentration of GBP over the ranges given in Table 1. 

**Table 1:** Summary of validation parameters calculated for the optimized and validated method

**Validation Parameter **	**Result **
Equation	Y = 0.0061X + 0.4004
SD of slope(S_b_)	0.0001
SD of intercept(S_a_)	0.0001
r^2^	0.9967
LOD(mg/L)	0.25
LOQ(mg/L)	0.8
Linear range in water (mg/L)	10 – 90
Linear range in plasma (mg/L)	0.8 – 10
Inter day precision ( RSD% , n=6 )	
10 (mg/mL)	1.2
90 (mg/mL)	0.1
Intraday precision ( RSD% , n=6)	
10 (mg/mL)	2.9
90 (mg/mL)	0.3
Accuracy ( Error% , n=6 )	
10 (mg/mL)	5.5
90 (mg/mL)	0.4
Recovery%	
10 (mg/mL)	85.5%
90 (mg/mL)	91.1%
Interference (matrix)	0.03-1.2%

The Table also shows the detection limits and the results of the statistical analysis of the data, such as the slopes, the intercepts; the correlation coefficients obtained by the linear least squares› treatment of the results along with standard deviation of the slope (S_b_) and intercept (S_a_) on the ordinate. The good linearity of the graph and the negligible scatter of the calibration points are clearly evident by the values experimental of the correlation coefficients and standard deviations. 

**Figure 5:**
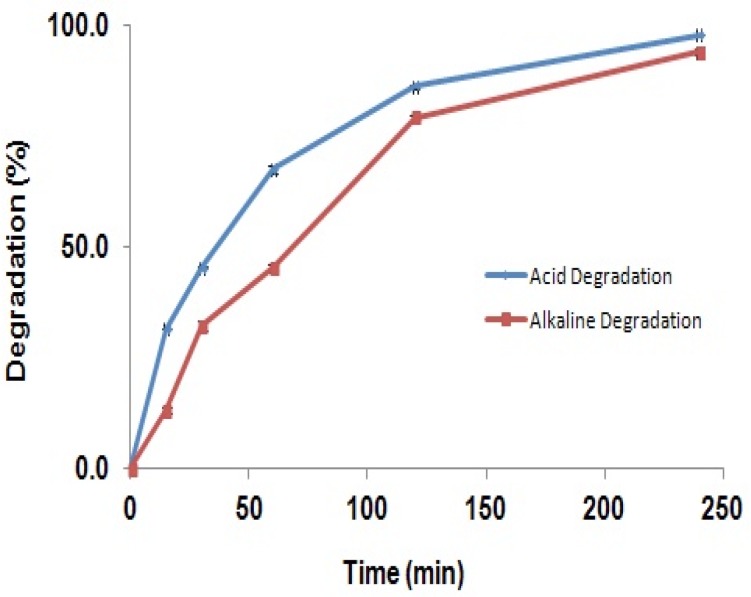
Degradation of GBP solution refluxed in acidic and alkaline media (Degradation percent of GBP in acidic (blue line) and alkaline (red line) solutions at 15 to 240 min).

The selectivity of the method was investigated by observing any interference encountered from the formulation matrix and degradation products that may be produced in chemical stress conditions. The degradation percent of GBP solution refluxed in acid and alkaline media at different times have been shown in Figure 5.

The figure shows that the degradation of the GBP in acidic or alkaline solutions was increased consecutively by time so that after 4 hours more than 90% of the drug was degraded, while the shapes of the spectra were not changed. It can be concluded that GBP is degraded in strong acidic and alkaline solutions; however, its degradation products are not interfering with the parent molecule. Results suggest this method can be used in stability studies as a stability indicating method. Furthermore, probable interference of excipients of the capsule mass such as talc, lactose, starch, microcrystalline cellulose, gelatin, and magnesium stearate was investigated. Results in Table 1 showed that, excipients may have interference of 0.03 to 1.2% related to the concentration of GBP. 

Results of the stability of the GBP standard solutions and sample preparations stored in ambient temperature showed that the solutions can be used until two weeks after preparation and the stability of the complex is long enough to read the absorption of the solutions during 4 h. 

*Pharmaceutical dosage form analysis*

The applicability of the proposed method was examined by the assay determination of GBP in bulk drug and capsules. GBP contents were shown to be 98 ± 2.3% both in bulk drug and capsules. Results indicated that this method could be utilized for the analysis of GBP in bulk and capsule formulation. 

*Plasma sample analysis*

It was shown that plasma concentrations of GBP after administration of a single dose of 900 mg to adult male volunteers are in the range of 0.8 to 10 mg/L. Different solid phase systems were tried for this purpose and C18 cartridges were chosen. By this way GBP was at least ten times concentrated and adequately separated from closely eluting endogenous amino acids existing in the biological samples and following this procedure, the samples were derivatized with vanillin reagent without further extraction procedure for derivatized reagents. LOQ of the proposed method after preconcentration with solid-phase extraction process was estimated to be 0.8 mg/L, therefore, it is postulated that it can be used for the determination of GBP in plasma in therapeutic drug monitoring. Drug-free plasma sample analysis by the developed method revealed maximum interfering effect of plasma components is less than 1.6% in the overall process. Results of determination of GBP in plasma samples indicates that preconcentration and clean up by SPE enables this method as a rapid, inexpensive, and reliable for the analysis of GBP in plasma levels as low as 0.8 mg/L. 

## Conclusion

Developed spectrophotometric method was found to be simple, accurate, precise, and linear across the analytical range. The method was selective for the determination of gabapentin in bulk, pharmaceutical formulations, and plasma. The method may be used to assess the quality of various gabapentin dosage forms by assaying for potency, content uniformity, dissolution and accurately monitoring the concentration in plasma. 
